# Acute renal failure caused by pheniramine maleate induced rhabdomyolysis: An unusual case

**DOI:** 10.4103/0972-5229.60176

**Published:** 2009

**Authors:** G. Paul, P. Sood, B. S. Paul, S. Puri

**Affiliations:** **From:** Critical Care Team, Dyanand Medical College and Hospital, Ludhiana, India; 1 **From:** Department of Neurology, Dyanand Medical College and Hospital, Ludhiana, India; 2 **From:** Department of Medicine, Dyanand Medical College and Hospital, Ludhiana, India

**Keywords:** Antihistamine overdose, pheniramine maleate, rhabdomyolysis

## Abstract

Antihistamines are easily available over-the-counter medications, which are frequently involved in overdoses. The usual course is accompanied by the anticholinergic effects of these agents. We report a case of a suicide attempt in a young male, where ingestion of antihistamine pheniramine maleate was complicated by nontraumatic rhabdomyolysis and oliguric acute renal failure. Rhabdomyolysis and acute renal failure is a rarely reported but potentially serious complication among patients who present to the emergency after intentional overdoses making recognition and prompt intervention essential. We also describe the potential mechanism of muscle injury in antihistamine overdose.

## Introduction

Antihistaminic, pheniramine maleate, is an alkylamine derivative and easily available over-the-counter drug popularly used for the treatment of allergy and cold symptoms. We report a case of rhabdomyolysis complicated by oliguric acute renal failure associated with high dose of pheniramine maleate ingestion for suicidal purpose.

## Case Report

A 24-year-old man was brought to emergency department with an acute febrile illness associated with altered sensorium. The patient was restless, agitated, and disoriented. Except for rhinorhea, the past medical history was unremarkable. There was no history of substance abuse or drug overdose.

Clinical examination showed an axillary temperature of 98°F, blood pressure of 130/76 mm Hg, pulse rate of 120/min, respiratory rate of 20/min, and pulse oximetry showed an oxygen saturation of 96%. Systemic examination showed dry mucosa and 4 mm pupils reacting to light. His cardiac, respiratory and abdominal examinations were normal. Neurological evaluation revealed a disoriented man with normal motor findings. There were no signs of meningeal irritation. Laboratory data revealed a normal haemogram and blood biochemistry. ECG showed no conduction disturbances but only sinus tachycardia. CSF study and MRI brain were normal. Urine toxicology screen was negative for benzodiazepines and opiates.

Patient was shifted to the critical care unit for observation. Few hours later, he developed cola colored urine. It was only then his relatives admitted that he had consumed 17 tablets of Avil 50 (pheniramine maleate 45.3 mg/tab) 8 hours prior to admission. Patient used to take this tablet for symptoms of rhinorhea. There was no other history that could explain his high coloured urine.

Laboratory data on the following day showed albumin 5.3 g/dl, total bilirubin 0.7 mg/dl, alkaline phosphatase 75 U/L, ALT 1471 IU/L, AST 238 IU/L, and lactate dehydrogenase 412 IU/L. Creatine phosphokinase (CPK) was 14114 IU/L. Urine was negative for myoglobin.

Clinical and laboratory parameters along with the history were suggestive of pheniramine-induced rhabdomyolysis. Treatment was started to enable forced alkaline diuresis but he still developed oliguria from fourth day onward. In view of rising blood urea and serum creatinine levels, the patient was taken up for hemodialysis. Oliguric phase persisted for 2 weeks, requiring alternate day hemodialysis. During third week, serum creatinine and CPK gradually started decreasing, with all abnormal lab findings normalizing and urine output gradually improved. The patient was discharged on the 23rd day.

## Discussion

Pheniramine maleate, an alkylamine, belongs to first generation of centrally acting H1 receptor antagonists. Alkylamine derivatives are among the most potent antihistaminics producing more CNS stimulation and less drowsiness. They also have potent competitive inhibition of muscarinic receptors causing anticholinergic side effects. Pheniramine is associated with a relatively high incidence of seizures (30%). It is one of the easily available over-the-counter drugs, and hence has high potential for misuse. The maximum dose of 3 mg/kg per day should not be exceeded. Our patient consumed approx. 770 mg. Overdose above the maximum tolerated dose led to appearance of muscarinic features and rhabdomyolysis. Patients who use antihistaminic chronically develop tolerance to the psychomotor performance and sedative effects due to autoinduction of hepatic enzymes,[1] as in this case.

Rhabdomyolysis is the rapid breakdown of *skeletal muscle* and leakage of myocyte contents into extracellular compartment caused by traumatic (physical) or nontraumatic (chemical or biological) factors. Drugs and alcohol have been responsible in 81% cases of nontraumatic rhabdomyolysis.[2] Rhabdomyolysis is commonly associated with myoglobinuria and can lead to potentially lethal complication like acute renal failure (ARF), the mechanism of which includes obstruction of tubular lumina by myoglobin casts, nephrotoxicity due to ferrihemate (breakdown product of myoglobin), and diminished glomerular filteration rate. Among patients with nontraumatic rhabdomyolysis, 33% develop ARF and 15% of them require dialysis.[3]

Hampel *et al*. firstly reported rhabdomyolysis secondary to antihistaminic toxicity leading to renal failure associated with myoglobinuria and CPK value of 30,000 U/L.[4] There are limited data in literature regarding rhabdomyolysis due to antihistaminic overdose. Only 11 cases of doxylamine and two of diphenhydramine toxicity have been reported to be associated with rhabdomyolysis.[5–7] CPK levels in these cases ranged from 597 to 78,750 U/L. ARF requiring hemodialysis occurred in only one of these patients.[8] No case report of rhabdomyolysis with pheniramine has been reported to the best of our knowledge. Our patient had a CPK level of 14,110 U/L at diagnosis and developed oliguric renal failure on fourth day onward requiring hemodialysis.

Drug-induced rhabdomyolysis can be due to primary or secondary myotoxic effect.[9] Primary effect is a direct insult on the skeletal myocyte function and an imbalance between the production and use of ATP in the muscle fiber. It is hypothesized that drug may exert a direct toxic effect on sarcolemma, increasing sodium permeability that disrupts calcium homeostasis, leading to alteration in intracellular energy sources and enhancement of intracellular proteolytic enzyme activity. The cell will then undergo progressive injury causing rhabdomyolysis. Secondarily, antihistamines by impairing the CNS can cause rhabdomyolysis by pressure-induced ischemia due to prolonged immobilization. Seizures due to overdose can also contribute to muscle breakdown as previously reported in case series, but there is an absence of seizure activity or prolonged immobilization in this patient.

Rhabdomyolysis is most reliably diagnosed by elevated levels of creatine phosphokinase in the blood (>five times). Initial and peak CPK levels have a linear relationship with the risk of ARF. Myoglobinuria may precede and resolve prior to an increase in CPK due to a short half-life of 1–3 hours. Therefore, a negative orthotolidine reaction does not rule out rhabdomyolysis, and myoglobin is found in urine only in 57% of patients during initial stage of rhabdomyolysis.[10] Other metabolic abnormalities include metabolic acidosis, elevated lactate dehydrogenase, amino transferases, creatinine, and urea. All except myoglobinuria were present in this case also.

Diagnosis of this patient was based on clinical picture and high CPK levels. In the absence of trauma, infectious diseases, muscular compression, convulsions, or metabolic disorders, rhabdomyolysis in this patient was attributed to pheniramine and was supported by high CPK levels. As other potential causes of the patient's ARF were ruled out, it was thought to be rhabdomyolysis.

Treatment of antihistamine-induced rhabdomyolysis requires recognizing the toxidrome associated with antihistamines, aggressive volume replacement, careful monitoring of electrolytes and renal function, forced alkaline diuresis, and hemodialysis if these conservative measures fail. A delay in establishing the diagnosis in this case likely contributed to the severity of renal failure.

We thought it would be interesting to report this case because easy availability of antihistamines increases the incidence of potential overdose. It may be one of the causes of coma with rhabdomyolysis leading to renal failure. Hence, depending only on laboratory findings without emphasis on history and clinical presentation may miss the diagnosis of antihistaminic poisoning leading to a medico-legal pitfall. More studies would be required to delineate the exact pathophysiology of rhabdomyolysis caused by antihistamines apart from the proposed primary and secondary effects. Antihistamine-induced rhabdomyolysis is a life-threatening condition, but early recognition and institution of effective treatment may lead to good prognosis, as seen in our patient.
